# An organotypic slice culture to study the formation of calyx of Held synapses *in-vitro*

**DOI:** 10.1371/journal.pone.0175964

**Published:** 2017-04-18

**Authors:** Elin Kronander, Nicolas Michalski, Cécile Lebrand, Jean-Pierre Hornung, Ralf Schneggenburger

**Affiliations:** 1 Laboratory of Synaptic Mechanisms, Brain Mind Institute, School of Life Science, École Polytechnique Fédérale de Lausanne (EPFL), Lausanne, Switzerland; 2 Department of Fundamental Neurosciences, University of Lausanne, Lausanne, Switzerland; Virginia Tech Carilion Research Institute, UNITED STATES

## Abstract

The calyx of Held, a large axo-somatic relay synapse containing hundreds of presynaptic active zones, is possibly the largest nerve terminal in the mammalian CNS. Studying its initial growth *in-vitro* might provide insights into the specification of synaptic connection size in the developing brain. However, attempts to maintain calyces of Held in organotypic cultures have not been fruitful in past studies. Here, we describe an organotypic slice culture method in which calyces of Held form *in-vitro*. We made coronal brainstem slices with an optimized slice angle using newborn mice in which calyces have not yet formed; the presynaptic bushy cells were genetically labeled using the *Math5* promoter. After six to nine days of culturing, we readily observed large *Math5*—positive nerve terminals in the medial nucleus of the trapezoid body (MNTB), but not in the neighboring lateral superior olive nucleus (LSO). These calyx—like synapses expressed the Ca^2+^- sensor Synaptotagmin-2 (Syt-2) and the Ca^2+^ binding protein Parvalbumin (PV), two markers of developing calyces of Held *in vivo*. Application of the BMP inhibitor LDN-193189 significantly inhibited the growth of calyx synapses, demonstrating the feasibility of long-term pharmacological manipulation using this organotypic culture method. These experiments provide a method for organotypic culturing of calyces of Held, and show that the formation of calyx—like synapses onto MNTB neurons can be preserved *in-vitro*. Furthermore, our study adds pharmacological evidence for a role of BMP-signaling in the formation of large calyx of Held synapses.

## Introduction

The calyx of Held synapse of the auditory brainstem has become a model system to study the biophysical mechanisms of presynaptic function, because of its large presynaptic nerve terminal accessible to direct patch-clamp recordings [1–4]. There is also considerable recent interest in understanding how this large, and highly specialized synapse is formed initially [5–9], because understanding the mechanisms of synapse size specification promises to yield insights into how synaptic circuits are wired during brain development. Calyx of Held axons originate from a subtype of bushy cells in the ventral cochlear nucleus (VCN), which in cats and rats have been identified as globular bushy cells (GBCs) [10–12]. Axons grow out early and cross the midline at ~ embryonic day (E) 14 in rodents [13, 14]. At around birth, pre-calyceal axons establish small nerve terminals with MNTB principal cells [5, 7, 8, 15]. A few days later, a process of nerve terminal growth and elimination of competing inputs takes place, leading to the establishment of a single large, but still immature calyx synapse on each MNTB principle cell [7, 16]. Thus, at postnatal day P4—P5, the basis of the highly specialized 1:1 innervation between a single GBC, and an MNTB principal cell is laid down. Identification of the molecular factors which drive this initial calyx of Held growth program could provide useful insights into the mechanisms that guide the formation of specialized synaptic connections in the CNS.

To learn more about the molecular mechanisms of the specification of nerve terminal size in the mammalian CNS, it would be beneficial to capture the growth of calyx of Held nerve terminals using an organotypic slice culture model. This would allow one to apply long-term pharmacological—and shRNA based methods to investigate the molecular mechanisms of calyx growth. However, previous attempts to maintain calyces of Held in organotypic cultures have not been successful [17, 18]. Another study reported miniature EPSC recordings from putative MNTB neurons in an organotypic culture of newborn mice, but this culture was not characterized morphologically [19]. A recent study, using dissociated cultures of VCN and MNTB neurons showed that large, calyx-like nerve terminals can form *in-vitro* onto putative MNTB principal cells [20].

The aim of the present study was to develop an organotypic slice culture which can capture the initial growth of the large calyx of Held nerve terminals onto MNTB neurons. We used brains from newborn mice, in which bushy cells were genetically labelled under the Math5 promoter [21], to allow visualization and preservation of the bushy cell axons in slice cultures. This, together with optimization of the slice angle, has allowed us to develop an organotypic slice culture in which large calyx-type synapses develop *in-vitro*.

## Materials and methods

### Ethics statement

All experimental procedures with laboratory mice were approved by the Veterinary Office of the Canton of Vaud, Switzerland (Authorization #2063.3). Newborn mice were kept in the homecage with their mother; any unnecessary disturbance of the mice was avoided. A newborn mouse at a time (postnatal day 0 [P0]; P0 refers to the day of birth) was removed from the cage, and killed by decapitation without prior anesthesia (protocol approved by the Veterinary Office of the Canton of Vaud, Switzerland).

### Mouse lines

We used a *Math5*^*Cre*^ mouse line described previously (Atoh7tm3(cre)Gan line; ref. [22]), to allow for genetic labeling of bushy cells in the VCN [21]. We bred *Math5*^*Cre*^ mice with the Brainbow Tg(Thy1-Brainbow1.0)LLich reporter mice, called *Brainbow* mice hereafter [23]. Due to weak fluorescence, at least under our fixation conditions, we enhanced the YFP/CFP signal driven in a Cre-dependent manner from the Brainbow construct using an anti-GFP antibody (chicken anti-GFP, 13970, Abcam; see below for staining procedures). Thus, we could not take advantage of the combinatorial effect of the Brainbow construct, but this might be possible in future studies.

The breeding pairs of *Math5*^*Cre*^ x *Brainbow* mice were homozygous for each allele; it was thus not necessary to genotype mice before the preparation of organotypic cultures. For the preparation of organotypic slices, newborn male and female mouse pups of the above genotype were used at the day of birth (referred to as postnatal day 0, P0), or exceptionally at one day after birth (P1). In some cases, *Math5*^*Cre*^ mice were crossed with a Cre-dependent tdTomato reporter mouse line, tdTomato Gt(ROSA)26Sortm9(CAG-tdTomato)Hze (Ai9; [24], and tdTomato was visualized with a rabbit anti-RFP antibody (AbCam 34771, polyclonal, AB_777699, 1:500).

### Organotypic slice preparation

Organotypic slice cultures were prepared on hydrophilic cell culture membranes (PICMORG50, Millicell) according to the general procedures of Stoppini et al. 1991 (ref. [25]). All following steps were performed in a laminar flow hood for cell culture under semi-sterile conditions. A mouse pup at a time was killed by decapitation without prior anesthesia, the brain was carefully dissected out under a stereomicroscope, and quickly placed in cold dissection medium, which was composed of 1X MEM (11012–044, Gibco powder), supplemented by 145 mM Tris (C4H11NO3, Biosolve) and 29 mM Glucose (G7528 Sigma). After visual inspection of the ventral side of the brainstem under a stereomicroscope (brains were discarded if the ventral side showed signs of damage), the brain was placed with its ventral surface onto the stage of a tissue chopper platform (McIlwain). Thus, the blade of the tissue chopper entered the brainstem tissue from its dorsal side. No glue was used to fix the brainstem to the tissue chopper platform. The hindbrain was cut into coronal slices of 350 μm thickness using the tissue chopper. The sliced hindbrain was collected into a petri dish by gently washing it off the tissue chopper stage by dissection medium. The slices were carefully separated using fine forceps (#5) and a preliminary selection of 2–4 slices was made under visual inspection with a stereomicroscope (not equipped with fluorescence). A final selection was made using an inverted fluorescence microscope (Olympus CK40; mercury lamp excitation light source and eGFP or CY3 filter sets), by selecting 1–2 slices which showed YFP fluorescence in the area of the VCN (this step was performed outside the hood). Back in the hood, a cell culture membrane (PICMORG50, Millicell) was placed on a drop of dissection medium and the selected slices were transferred on top of the membrane. Excessive medium was removed until only a thin layer covering the slices remained. The inserts were transferred into a 6-well plate or 35 mm petri dish with 1 ml of freshly prepared, pre-heated and equilibrated culture medium (see below for composition). The slices were placed in an incubator (37°C and 5% CO_2_), and every second day, 500 μl culture medium (corresponding to ~ 50% of the total volume) were aspirated and replaced by fresh culture medium.

### Culturing medium

The culture medium was Neurobasal medium (12348–017, Gibco), supplemented with B27 (17504–044, Gibco; 1:100; one-half of the concentration recommended by the supplier), 2 mM L-Glutamine (25030–024, Gibco; 1:100), Penicillin-Streptomycin (15140–022, Gibco; 1:100). We increased the extracellular K^+^ concentration by adding 25 mM KCl [26], using a KCl stock solution of 2.5 M.

### Rotation angle during section preparation

We found that organotypic slices in the coronal plane typically did not show a preserved MNTB and VCN in the same slice (S1 Fig). We therefore introduced an angle for the preparation of the coronal slices, by turning the coronal plane of the brain around a vertical axis lying in the brain's midline, using the rotating platform of the tissue chopper. The direction of the turn was decided after visual inspection, with the aim to use the better—preserved VCN for culturing. In preliminary experiments, we tried various slice angles (~ 5–30°) and found the most useful one to be ~15°.

### Success rate and number of usable organotypic slice cultures

In one culturing session, we typically prepared organotypic slices from 6–10 newborn mice of a given litter. The success of each organotypic culture was analyzed *post-hoc* by confocal imaging of the cultures, enhanced by immunohistochemistry (see below). Successful organotypic slice cultures which could be used for imaging analysis of calyx size were defined as follows: 1) A clear VCN area with Math5 (GFP)—positive neurons was visible, 2) an ipsilateral, or an ipsi- and contralateral MNTB was present, 3) Math5—positive axons were present close to the MNTB, and 4) no abnormalities or obvious signs of tissue damage were present.

For the data reported in this study, we obtained n = 49 successful organotypic slices, using a total of n = 111 mouse pups. Thus, the success rate was roughly 50%. The above number does not include the initial attempts with varying or no slice angles (S1 Fig), or trials in which we cultured the slices for less than 6 days.

### Pharmacological manipulation with LDN-193189

Pharmacological manipulations of the organotypic cultures were done in strictly pairwise experiments. The ~ 6–10 slice preparations obtained from one litter (see above) were separated in two groups; one group was cultured in the presence of LDN-193189 (5 μM; added from a 10 mM stock solution in DMSO; Stemgent); a control group was cultured with an equivalent amount of DMSO (1:2000 vol. dilution). With this arrangement, we aimed to obtain at least one usable organotypic slice for each condition from a given mouse litter, to allow for pairwise comparison within one day of organotypic slice preparation. LDN-193189 (or DMSO alone) was present from the beginning of culturing throughout 8 days *in-vitro* (DIV), the time point at which the slices were fixed and processed for immunohistochemistry.

### Immunohistochemistry

After 6, 8 or 9 days of culturing, the organotypic slice cultures were fixed with 4% paraformaldehyde (PFA) in PBS for 30 min and washed 3 times; then the cell culture membrane was cut to separate the organotypic slices. The individual slices were washed 3 × 10 minutes and permeabilized with 0.3% Triton X-100 in PBS 3 × 10 minutes, followed by a 1 hour incubation in the additional presence of 2% normal horse serum. The slices were then incubated with primary antibodies in the same buffer at 4°C for 72 hours. The slices were washed 3 x 10 min in PBS and 0.3% Triton X-100, and then incubated for at least 18 hours at 4°C with the secondary antibody. After washing 3 × 30 min in PBS the slices were mounted with fluorescence mounting medium (Dako) on superfrost plus slides with a 120 μm secure-seal spacer (ThermoFisher scientific) glued onto it. Slides were stored in darkness at 4°C between imaging sessions.

**Fig 1:**
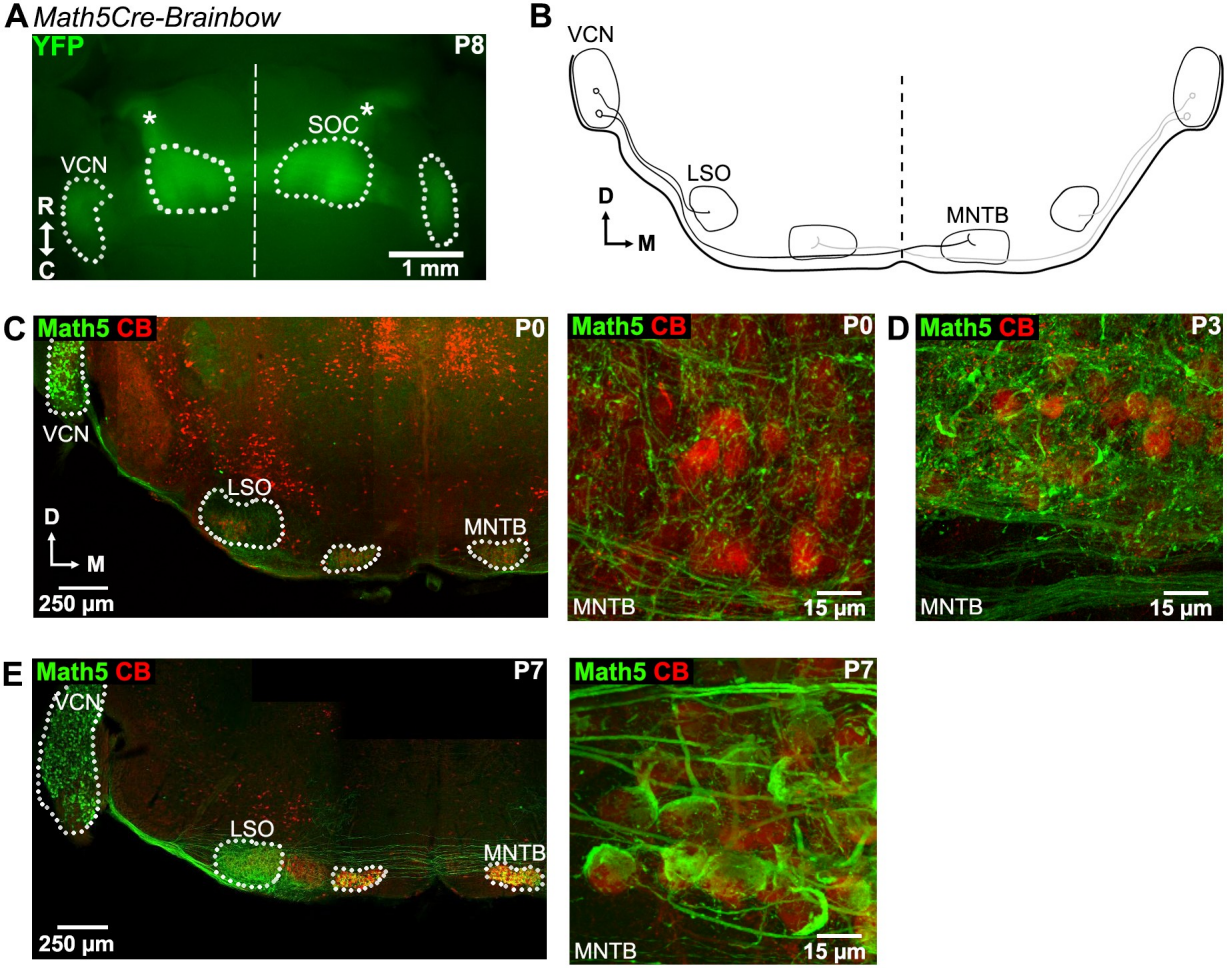
Visualization of bushy cell axons in *Math5*^*Cre*^ x *Brainbow* mice early postnatally. ***A***, Ventral view of the brainstem of a P8 *Math5*^*Cre*^ x *Brainbow* mouse. The YFP fluorescence of the Brainbow reporter was excited by 470 nm excitation light in a stereomicroscope equipped for fluorescence. The extent of the VCN and of the superior olivary complex (SOC) are outlined; the midline is indicated by the straight dashed line. Note the strongly YFP-positive ventral acoustic stria exiting the VCN, and the lateral lemniscus (star symbols). ***B***, Schematic overview of a coronal section of the mouse auditory brainstem. Bushy cells in the VCN projecting to the ipsilateral LSO and to the contralateral MNTB are indicated. ***C-E***, Confocal images of coronal sections of the auditory brainstem of *Math5*^*Cre*^ x *Brainbow* mice at three ages (C, P0; D, P3; E, P7). The sections were stained with an anti-GFP antibody (green channel; to detect the Cre-dependent YFP and CFP expression driven by the Brainbow reporter mouse; labelled "Math5"), and with an anti-Calbindin antibody as a marker for MNTB neurons (red channel). Note the presence of Math5—positive axons with few collaterals and no large nerve terminals at P0 (C, *right*); with more numerous collaterals at P3 (D), and with large calyceal nerve terminals at P7 (E, *right*).

For immunohistochemistry of brain sections (Fig 1), a P3 and a P7 *Math5*^*Cre*^ x *Brainbow* mouse were anesthetized with pentobarbital and transcardially perfused with fixative (4% PFA in PBS). The brain was dissected and post-fixed in the same fixative for 12–16 hours at 4°C. In the case of the P0 *Math5*^*Cre*^ x *Brainbow* mouse, the brain was dissected in ice-cold PBS and then immersion-fixed in 4% PFA at 4°C during 24 hours. Dehydration of the fixed brains, sectioning (30 μm mircotome sections), washing and antibody incubation (16–20 hours at 4°) was done according to standard methods.

### Antibodies

Organotypic slice cultures were stained with combinations of three (or sometimes two) antibodies to extract a maximum of morphological information from each slice. We always enhanced the YFP/CFP fluorescence driven by the Brainbow construct [23] with a chicken anti-GFP (Abcam 13970) antibody.

The following combinations of primary and secondary antibodies were used in each Figure:

Fig 1, primary antibodies: chicken anti-GFP (AB_300798; Abcam 13970; polyclonal; 1:1000) and rabbit anti- Calbindin D-28k (AB_10000340; Swant CB-38a; polyclonal; 1:500).

**Fig 2:**
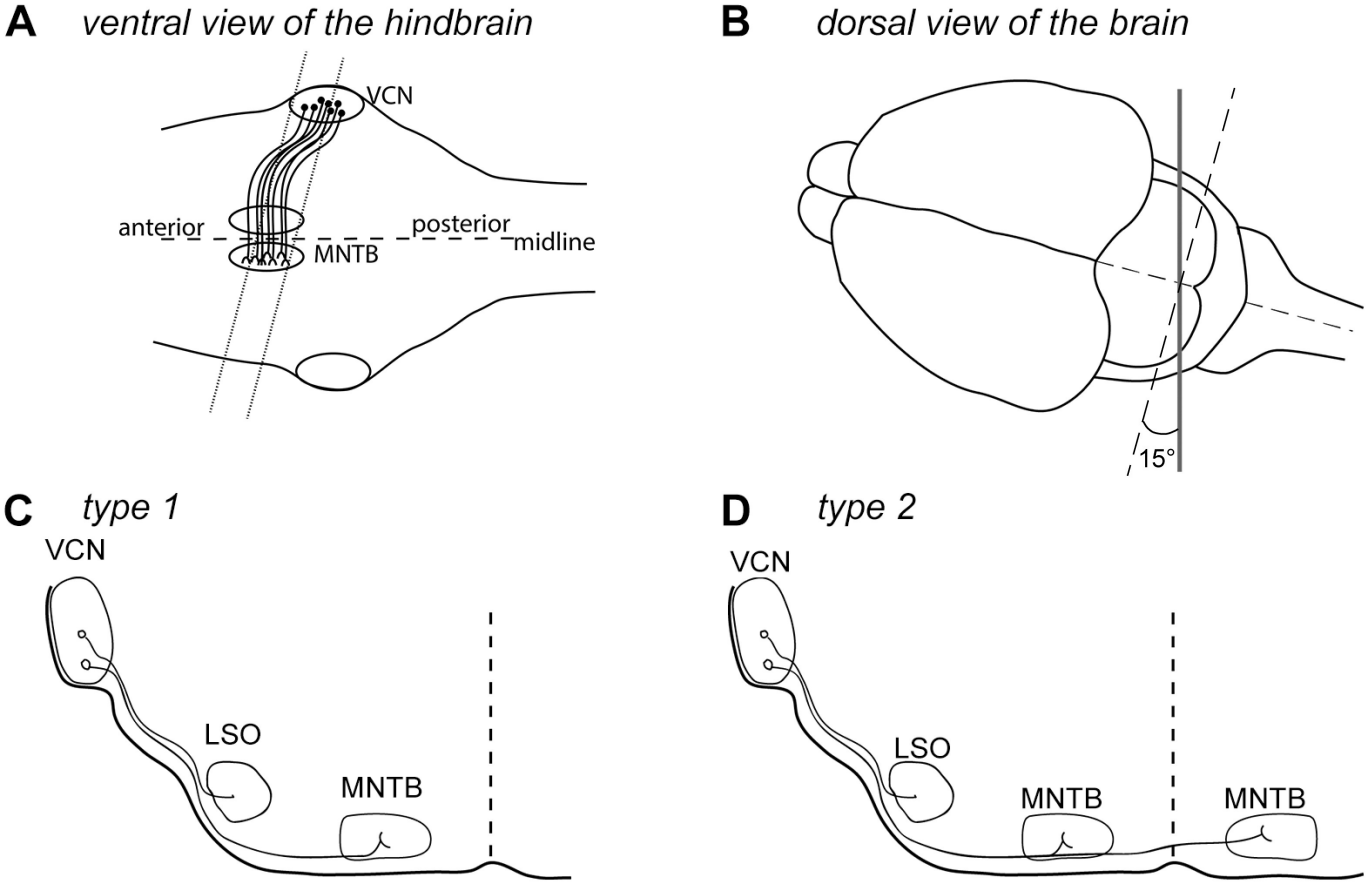
Modified angle during preparation of coronal hindbrain slices to preserve the VCN—MNTB connectivity. ***A***, Ventral view of the brainstem. Based on several observations with organotypic slice culturing of hindbrain tissue from newborn mice, we infer that the MNTB nuclei are located more anteriorly than VCN (see also Fig 1A). The dashed line indicates the desired slicing configuration using the 15° rotation. ***B***, Dorsal view of the slicing configuration. Grey line indicates the orientation of the tissue chopper blade. ***C*, *D***, scheme of type 1 and type 2 organotypic slice cultures that we observed.

Secondary antibodies: Alexa 488 goat anti-chicken (AB_142924; Invitrogen A11039); Alexa 647 goat anti-rabbit (AB_141775; Invitrogen A21245). Fig 2; no antibodies were used.

**Fig 3:**
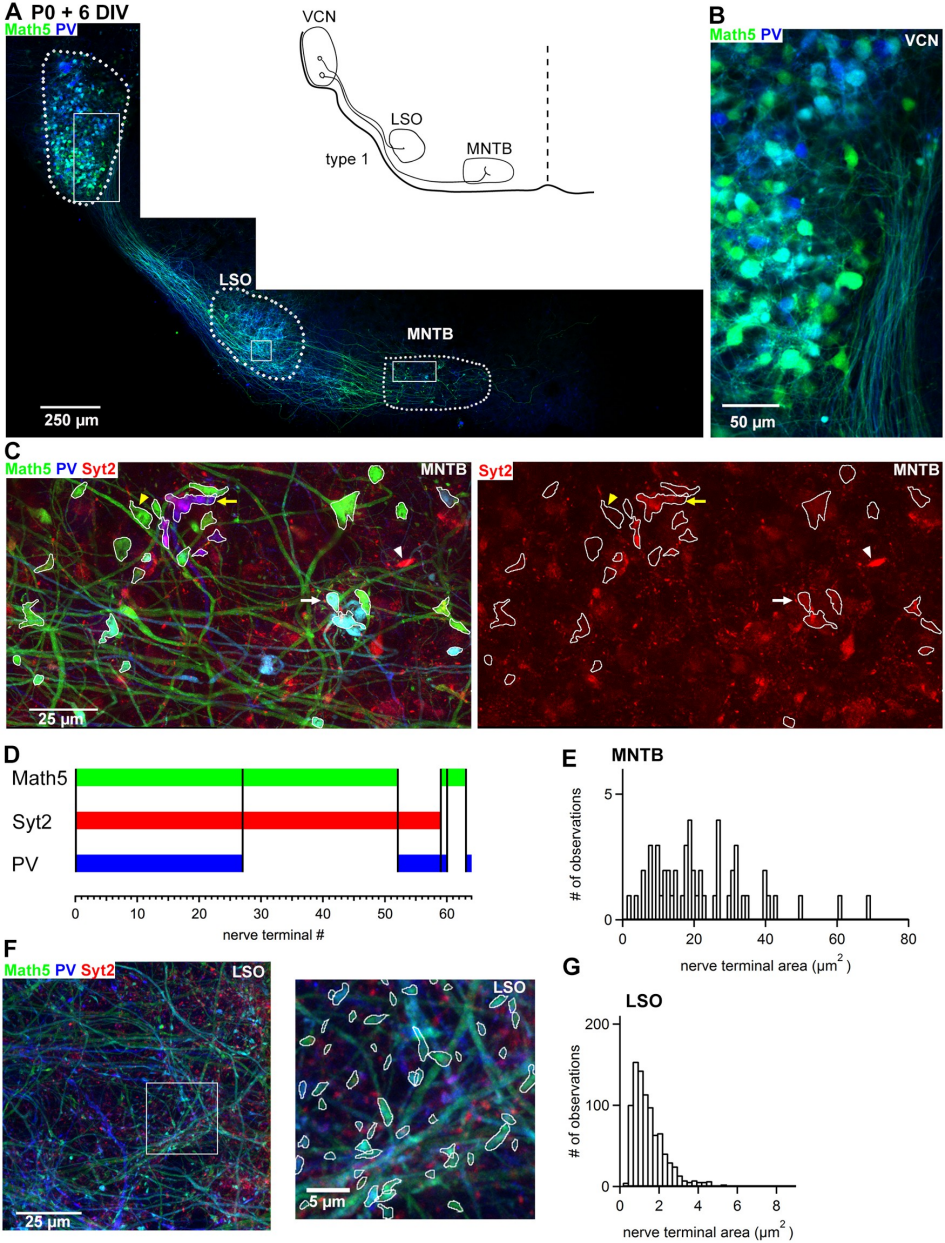
Math5- and Syt2-positive large nerve terminals are found in MNTB but not LSO neurons in organotypic slices. ***A***, Overview image of a slice culture prepared from a *Math5*^*Cre*^ x *Brainbow* mouse at P0, using a slice angle of 15° during preparation of the coronal slices (see Fig 2). After 6 days *in-vitro* the slice culture was fixed and stained with an anti GFP-antibody (green channel), an anti-PV antibody (blue channel), and an anti-Syt2 antibody (red channel). ***B***, VCN at a higher magnification. ***C***, Maximal intensity projection image (stack of 60 images; z step 0.5 μm) from within the MNTB area (boxed region in A); the right image shows the anti-Syt2 immunohistochemistry (red channel) in isolation. Large calyx-like terminals, captured on the level of their largest cross-section, are outlined in white. *White arrow*, example of a nerve terminal positive for Math5, Syt2 and PV; *white arrowhead*; nerve terminal positive for Syt2 alone; *yellow arrow*, nerve terminal positive for Syt2 and PV; *yellow arrowhead*, nerve terminal positive for Math5 alone. ***D***, Chart of the co-localization of the three markers Math5, Syt2, and PV in large calyx-type nerve terminals in the MNTB area resulting from the analysis of the slice culture shown in A—C. The presence, or absence of each fluorescent channel in each nerve terminal (abscissa) is indicated. Note that most Math5—positive nerve terminals express Syt2, and more than half also co-express PV. ***E***, Histogram of nerve terminal size found in the MNTB for this organotypic slice culture (n = 61 large nerve terminals), measured as shown in C. ***F***, Confocal image of the LSO subarea indicated in A with a box. Note the abundant axons which are either Math5—positive, or PV-positive, or stained by both antibodies, as well as numerous small bouton-like nerve terminals, most of which are Syt2-negative. The image on the right shows an inset at higher resolution. The masks drawn over Math5—positive nerve terminals are shown in white. ***G***, Histogram of nerve terminal size found in the LSO of this organotypic slice (n = 906 terminals analyzed in two image stacks as the one shown in *F*). Note the significantly smaller nerve terminal size in LSO (*G*) as compared to MNTB (*E*; p < 0.001).

**Fig 4:**
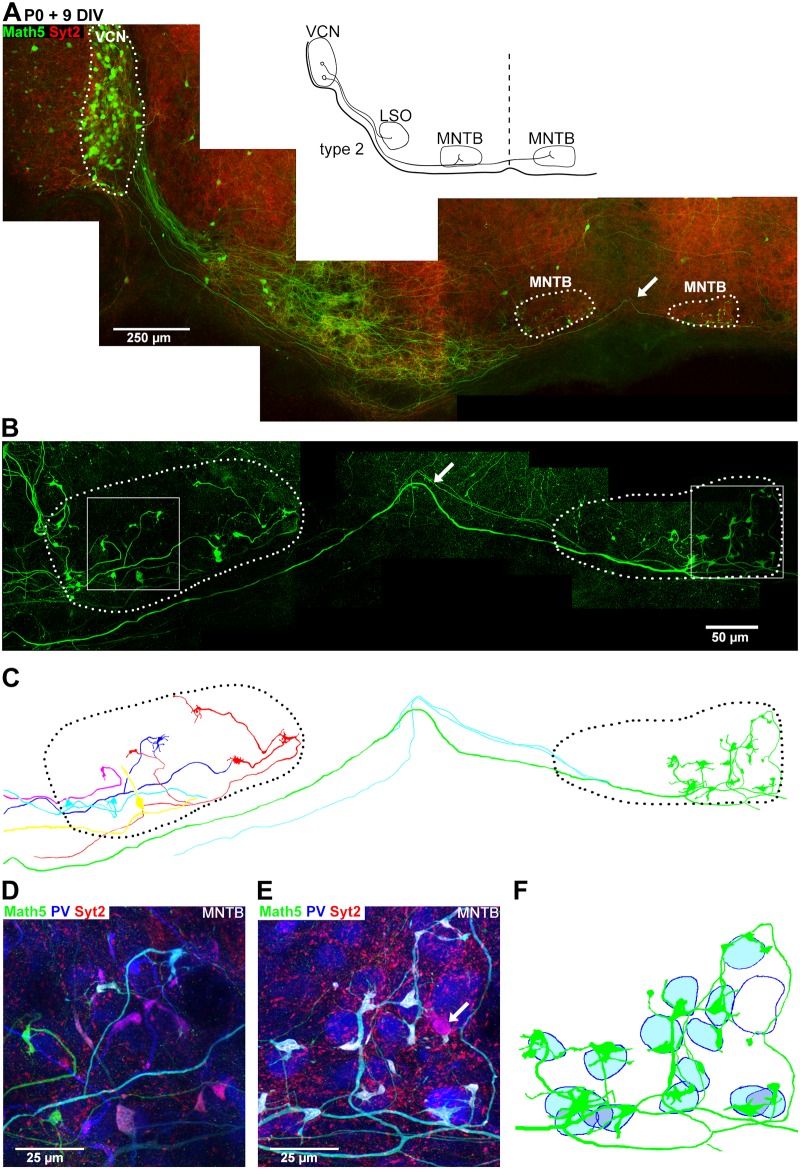
Example of a type 2 organotypic culture with preserved ipsi- and contralateral VCN-MNTB circuit. ***A***, Overview of a type 2 slice prepared with 15° slicing angle from a *Math5*^*Cre*^ x *Brainbow* mouse at P0. The organotypic slice was fixed after 9 days *in-vitro* and stained with an anti-GFP-antibody (green channel), anti-PV antibody (blue channel), and an anti-Syt2-antibody (red channel). The arrow indicates a midline-crossing axon. ***B***, Maximal intensity projection image (35 images; z step 0.5 μm) of the ipsilateral MNTB, midline region and contralateral MNTB for the GFP channel. Note the presence of Math5—positive axons which elaborate large nerve terminals. ***C***, Reconstruction of n = 5 ipsilateral axons (shown in red, blue, light blue, yellow, and pink), and of a large- diameter axon crossing the midline (green). This latter axon established large calyx—like synapses with n = 15 PV—positive neurons in the contralateral MNTB (see panel F for a more detailed view). Another two Math5—positive crossing axons (blue) did not establish large nerve terminals. ***D*, *E***, Maximal intensity projection images of all three fluorescent channels (31 images; z step 0.5 μm) of a region of the ipsi- and contralateral MNTB (D and E, respectively; see boxes in panel B). **F**, The reconstructed contralateral axon seen in C (green) at a higher resolution, and with the approximate positions of the postsynaptic neurons indicated. Note that this axon makes contact with n = 15 PV—positive MNTB principal neurons (filled blue circles). One PV—positive neuron (non-filled blue outline) received a large non Math5—positive nerve terminal from another source (see also panel E, white arrow).

Figs 3 and 4, Primary antibodies: chicken anti-GFP (AB_300798; Abcam 13970; polyclonal; 1:1000), rabbit anti-Parvalbumin (AB_10000344; Swant PV-25; polyclonal; 1:2000), mouse anti-Synaptotagmin 2 (AB_10013783; ZIRC znp-1; monoclonal; 1:500).

Secondary antibodies: Alexa 488 goat anti-chicken (AB_142924; Invitrogen A11039), Alexa 568 donkey anti-rabbit (AB_2534017; Invitrogen A10042) (for Fig 4: Alexa 405 goat anti-rabbit; AB_221605; Invitrogen A31556), Alexa 647 donkey anti-mouse (AB_162542; Invitrogen A31571). All secondary antibodies were used at a dilution of 1:300.

**Fig 5:**
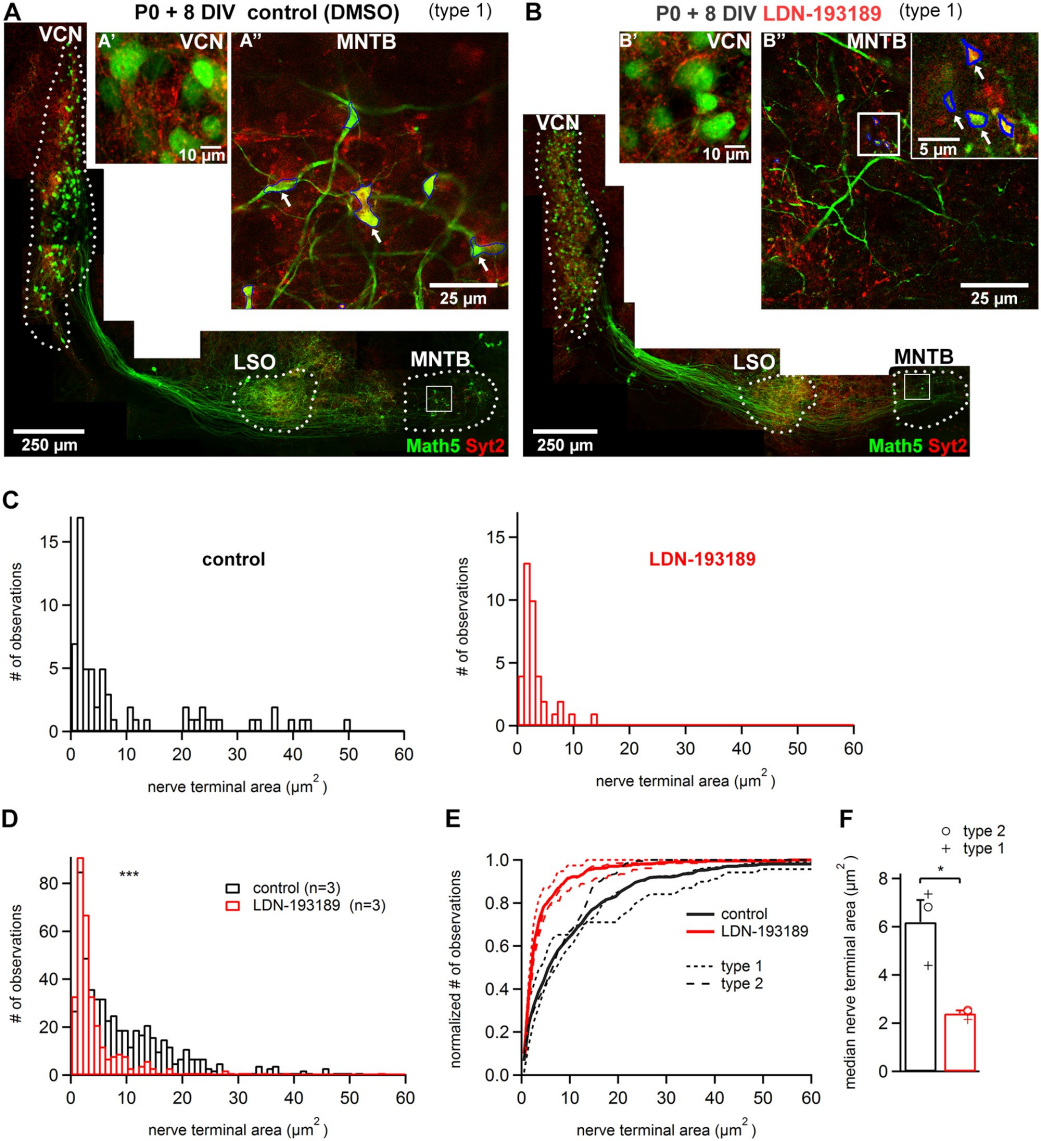
The BMP-SMAD inhibitor LDN-193189 reduces the size of calyces of Held developing in organotypic culture. ***A*, *B***, examples of organotypic cultures prepared at P0 from two *Math5*^*Cre*^ x *Brainbow* littermate mouse pups. The slices were cultured in the presence of DMSO alone (**A**) or 5 μM LDN-193189 (**B**), fixed after 8 days *in-vitro*, and stained with anti-GFP antibody, and anti-Syt2 antibody. ***A’*, *B’***, high-magnification confocal images on the level of the VCN. Note Math5- positive bushy cells visible under both conditions. ***A”***, ***B”***, confocal images on the level of the MNTB, in the areas indicated by rectangles in A and B. Note the presence of large, Syt2-positive nerve terminals under control conditions (A''), but the conspicuous absence of large nerve terminals under LDN-193189 at the same magnification (B''). The inset in B'' is a higher magnification image of B''. The blue lines in A'' and B'' are the nerve terminals outlines at their widest extension. ***C***, histograms of Syt2- and Math5- positive nerve terminal size of the entire MNTB region of the cultures shown in A and B (n = 68 and 38 nerve terminals for control, and for LDN-193189). ***D***, histogram of nerve terminal size from n = 3 organotypic cultures under each condition. Note the presence of large nerve terminals in the range of ~ 5–50 μm^2^, which are largely missing in slices cultured in the presence of LDN-193189. ***E***, Same data as in D, shown as cumulative histograms for each culture. Short- versus long dashed lines show results from type 1—and type 2 cultures, respectively; the fat black and red lines represent the average cumulative histograms across all cultures (n = 3 each). Note the clear shift towards smaller nerve terminal size for cultures made in the presence of LDN-193189. ***F***, Summary plot of the median nerve terminal size analyzed in individual organotypic cultures under each condition. The median nerve terminal size was significantly smaller in the LDN-193189 group as compared to the control group (p = 0.0019). Results from type 1 and type 2 cultures are shown by different symbols as indicated.

Fig 5: like Figs 3 and 4, but the PV-immunohistochemistry was omitted.

### Confocal imaging

Fixed and mounted organotypic slices were first visualized under a widefield microscope to check for GFP fluorescence and preservation of the VCN—MNTB projection. Suitable slices were then imaged in detail with a confocal microscope (LSM 710, Zeiss). For this, we first made an overview image with 10x, 0.3 NA, or 20x, 0.8 NA air objectives (Zeiss) in which various fields of view at a single focal depth were acquired and stitched together. The VCN, LSO and especially the MNTB areas were then imaged in more detail with 40x, 1.3 NA (or 63x, 1.4 NA) oil immersion objectives (Zeiss). For this, confocal image stacks of 1024 × 1024 pixels, pixel size 0.0992 μm, and Δz value of 0.5 μm, were acquired. We could typically image to a depth of ~ 40 μm into the slice. The thickness of organotypic slices after 6–9 days of culturing were ~ 100–150 μm as estimated by using a two photon microscope and focusing to the bottom of the slice.

### Image analysis

Confocal images were analyzed in ImageJ (Fiji toolbox; ImageJ 1.51 g). For display purposes, the brightness and contrast were sometimes adjusted in the entire image using image J or Adobe Photoshop. To analyze the size of nerve terminals, we used high-resolution confocal image stacks (see above) and identified the largest cross-section of a given Synaptotagmin-2 (Syt2)—and Math5-positive nerve terminal by eye. The nerve terminal was then outlined with the freehand tool of Fiji [27] to measure the synapse cross-sectional area (Figs 3 and 5). Average values of nerve terminal size were reported as mean ± S.E.M. In the analysis of Fig 5, we attempted to quantify all Math5—and Syt2—positive nerve terminals. The statistical significance of the different distributions of synapse size was assessed with a non-parametric Mann Whitney test, due to the non-normal distribution of the samples.

Tracings and reconstructions of GFP—labelled axons in confocal image stacks (Fig 4C and 4F) were done with the Simple Neurite Tracer plug-in of ImageJ [28]. Axons were traced by hand, and at the position of thickenings (putative nerve terminals), the fill-out function was used.

## Results

### Math5 genetically labels developing bushy cell axons

To develop an organotypic slice culture model of the calyx of Held synapse, it would be helpful to label presynaptic bushy cells and their axons genetically, to enable *post-hoc* visualization of bushy cell axons in the organotypic culture. It was shown previously that a *Math5*^*Cre*^ transgenic mouse line drives Cre-expression specifically in bushy cells of the VCN, comprising both GBCs and spherical bushy cells, SBCs [21]. We therefore used a *Math5*^*Cre*^ knock-in mouse line [22], crossed with the Brainbow reporter mice in most experiments [23], to genetically label bushy cells and their axons. We first verified whether the *Math5*^*Cre*^ line used here drives reporter gene expression sufficiently early and strong to allow for labelling of bushy cell axons at around birth.

In whole mounts of the mouse brainstem at P8, we observed YFP fluorescence driven in a Cre-dependent manner from the Brainbow construct in the VCN, as well as the axonal tract of the ventral acoustic stria emanating from the VCN and entering the superior olivary complex (SOC; Fig 1A). The lateral lemniscus was also YFP—positive (Fig 1A, star symbol), consistent with bushy cell axons that continue to the nuclei of the lateral lemniscus and to the inferior colliculus [14, 29].

We next used coronal sections (Fig 1B) and immunohistochemistry to label the transgenically expressed YFP and CFP reporter genes with an anti-GFP antibody (Fig 1C–1E; green channel; see Materials and methods). In addition, we used an anti-Calbindin antibody, which within the MNTB labels postsynaptic neurons selectively [30, 31]. On postnatal day 0 (P0) (day of birth), the reporter genes were well expressed, and the VCN showed strong YFP/CFP expression as detected with the anti-GFP antibody (Fig 1C left), indicating that the *Math5* locus was activated in neurons of the VCN earlier than P0. In what follows, we will refer to the YFP/CFP expressing cells as Math5—positive cells. In the MNTB, thin Math5—positive axonal structures but no big terminals were seen at P0 (Fig 1C, right). At P3, some larger axonal terminals close to the somata of MNTB neurons had developed (Fig 1D). At P7, large Math5—positive calyx of Held terminals were clearly visible in the MNTB, and the fiber tract emanating from the VCN was strongly labeled (Fig 1E). In further immunohistochemical stains at P9—P12, we found that 92 ± 4% of all calyx of Held nerve terminals, were Math5—positive (n = 5 mice; n = 842 calyces), in good agreement with a recent study [32]. Taken together, the clear expression of YFP/CFP in bushy cells axons in these *ex-vivo* preparations suggests that the *Math5*^*Cre*^ mice can be used to label bushy cell axons early postnatally. In addition, the labelling of the pre-calyceal and axonal structures under the Math5 promoter recapitulated the normal development of calyces of Held, with a clear absence of calyx nerve terminals at P0 [5, 7, 8, 15].

### Math5-positive large nerve terminals in the MNTB of optimized slice cultures

We next developed an organotypic slice culture of the calyx of Held, with the main aim to capture the initial growth of the calyx of Held terminals *in-vitro*. We used brainstem tissue from *Math5*^*Cre*^ x *Brainbow* mice at the day of birth (P0; see Materials and methods), an age at which large calyx of Held synapses have not yet formed *in-vivo* (see above). Transverse slices of 350 μm thickness were cultured according to the general methods of Stoppini et al. 1991 [25], using elevated K^+^ concentrations in the medium [26]. At the end of the culturing period (usually, 6–9 days *in vitro*), the organotypic slices were fixed, stained with antibodies and imaged with a confocal microscope (see Materials and methods).

In our first attempts, we could observe Math5—positive neurons in the lateral part of the slice, identifying this region as the VCN. We also observed a putative LSO which received Math5—positive axons forming small, VGluT2—positive nerve terminals. However, we could not observe a MNTB region, and Math5—positive axons did not extend more medially than the LSO area (S1 Fig).

A plausible explanation for the absence of the MNTB nucleus, and of bushy cell axons towards the midline in our preliminary preparations, is that the VCN is located more caudally than the MNTB (Fig 2A). To preserve the VCN-MNTB connection, we introduced a slicing angle of approximately 15° during the preparation of the tissue sections, turning the coronal plane of the brain around a vertical axis in the brain's midline (Fig 2A and 2B; and see Materials and methods). Because of the slicing angle, we expect to obtain only a single preserved VCN, with however improved connection to the MNTB area (Fig 2A, grey lines). *Post-hoc* confocal imaging showed that in many organotypic cultures (n = 28 / 49 of all successful preparations with a 15° angle), only the MNTB nucleus *ipsilateral* to the targeted VCN was maintained; these are referred to as "type 1" slice cultures. In other cases, "type 2" slice cultures comprising both an ipsi- and a contralateral MNTB were obtained (n = 21 / 49 successful organotypic cultures; see schemes in Fig 2C and 2D). The outcome of whether an organotypic slice would be type 1 or—2 could not be determined beforehand, but was assessed *post-hoc*.

Fig 3 shows an example of a type 1 slice culture maintained for 6 days *in-vitro*, and then fixed and stained with antibodies against GFP (to visualize Math5—positive cells and their axons), anti—Parvalbumin antibody (PV; a marker for bushy cells and MNTB neurons [31]) and anti—Syt2 antibody (to label calyces of Held [33, 34]). In this culture, the VCN, and the ventral acoustic stria could be clearly identified based on the Math5—positive cell bodies and emanating axons (Fig 3A and 3B). The morphology of the cells in the VCN (Fig 3B) was consistent with bushy cell morphology, with a rounded or oval cell body and a single primary dendrite [35, 36]. In the region ventro-medial to the VCN, we found many Math5—positive axons innervating the LSO area, where they elaborated small bouton-like terminals (see below; Fig 3F). More medially to the LSO, we observed an ipsilateral MNTB nucleus (Fig 3A). After longer culturing periods (9 days *in-vitro*; see Fig 4), these neurons expressed PV, consistent with the developmental expression onset of PV in MNTB principal cells at ~ P8 [31]. Thus, these neurons are most likely MNTB principal cells.

Within the MNTB, we observed Math5—positive axons which formed large nerve terminals, often expressing the calyx marker Syt2 (Fig 3C). About half of all Math5—and Syt2—positive nerve terminals expressed PV (Fig 3D; see white arrow in Fig 3C for an example), consistent with the expression onset of PV in calyces of Held nerve terminals at ~ P5—P6 *in-vivo* [31]. We also observed some large Syt2- and PV- positive nerve terminals which were not Math5—positive (Fig 3C, yellow arrow; 8/64 large nerve terminals; see Fig 3D). This is consistent with the *in-vivo* finding that not all calyces are Math5—positive (see above, and ref. [32]). Other combinations, like large Math5—positive nerve terminals without Syt2 (Fig 4C, yellow arrowhead), represented a small fraction of all nerve terminals (Fig 3D). These morphological observations suggest that large, Math5—and Syt2—positive nerve terminals are calyces of Held.

In order to measure the size of the nerve terminals, we identified the largest cross-section area of nerve terminals positive for both Syt2- and Math5 in confocal image stacks, and then drew this area by hand (white outlines in Fig 3C). The resulting distribution of nerve terminal areas was rather broad, with an average of 22.3 ± 1.9 μm^2^ for this culture, and with some very large nerve terminals of ~ 60 μm^2^ (Fig 3E).

To verify whether large nerve terminals are formed specifically in the MNTB as we would expect, we next quantified the size of putative nerve terminals made by the Math5—positive axons in the LSO (Fig 3F). We found no Math5—positive axons with large nerve terminal structures in the LSO (Fig 3F); the average size of the Math5—positive nerve terminals in the culture shown in Fig 3 was only 1.35 ± 0.03 μm^2^ (Fig 3G). This value was more than an order of magnitude smaller than the size of Syt2- and Math5—positive nerve terminals in the MNTB of the same culture (p < 0.001). This suggests that the propensity of GBC axons to form large calyces in the MNTB, and of SBC axons to form small synapses in the LSO, is maintained in slice culture. Of note, the large nerve terminals which we observed in the MNTB after 6 days *in-vitro* must have formed during the time in culture, because at P0, the day of culturing, calyces of Held are not yet present *in-vivo* (see Fig 1C) [5, 7, 15]. Thus, an optimized slice culture allows us to observe large calyx—like nerve terminals which have formed *in-vitro*.

### Math5-positive large nerve terminals form both ipsi- and contralaterally

Fig 4 shows the results from another slice culture in which both the ipsi- and the contralateral MNTB were preserved ("type 2" slice culture). Numerous Math5—positive bushy cells of the VCN sent axons into the ventral acoustic stria (Fig 4A). At the midline, a large-diameter axon crossed over to innervate the contralateral MNTB (Fig 4A, arrow; Fig 4B). In both the ipsi- and the contralateral MNTB, there were large diameter Math5—positive axons that frequently widened into large nerve terminals expressing both Syt2 and PV (Fig 4D and 4E; white in the overlay). In addition to the large Syt2—positive terminals, small Syt2—positive terminals were sometimes observed; these seemed to be of non-bushy cell origin since they were not Math5 positive (Fig 4E).

We next reconstructed the individual axons and their nerve terminals, using the GFP immunofluorescence signal in confocal image stacks (see Material and Methods). This revealed that all large Math5—positive nerve terminals found in the contralateral MNTB of this culture belonged to a single midline—crossing axon (Fig 4B, arrow; Fig 4C). This axon made contact with n = 15 PV—positive MNTB neurons contralaterally (Fig 4F), but it did not establish synapses in the ipsilateral MNTB. In the ipsilateral MNTB, we reconstructed n = 5 axons which elaborated large nerve terminals; each of these axons formed between n = 1–4 large nerve terminals on different postsynaptic neurons. These axons clearly arrived in the MNTB from the ipsilateral side, suggesting that GBC axons which might have been severed close to the ipsilateral MNTB during the culturing procedure, have the capacity to re-organize and to form nerve terminals ipsilaterally. Furthermore, the finding that single axons can readily make more than one calyx—like nerve terminal in organotypic culture, especially under conditions of axon paucity on the contralateral side (Fig 4C and 4F), suggests that competition between axons contributes to the 1: 1 connectivity seen *in-vivo* at the calyx of Held synapse (see Discussion).

Taken together, the morphological observations in Figs 3 and 4 reveal the presence of large, Syt2—positive nerve terminals formed by bushy cell axons onto MNTB neurons in organotypic slice cultures. In the LSO, only small nerve terminals of Math5—positive axons were seen. We did not observe obvious differences between type 1 and type 2 slice cultures regarding the morphology of large calyx—like synapses, or between calyx—like synapses formed ipsi—versus contralaterally (Fig 4C). This indicates that in the absence of a contralateral VCN, large nerve terminals can form on both the ipsi- and the contralateral MNTB. These observations suggest that in organotypic culture, bushy cell axons form large calyx—like synapses specifically onto MNTB neurons.

### A BMP-receptor inhibitor leads to significantly smaller calyx-like nerve terminals *in-vitro*

We next tested whether organotypic slice cultures of the developing calyx of Held can be used for long-term pharmacological experiments, using the size of Math5—and Syt2—positive nerve terminals as read-out. Previous genetic evidence has suggested that BMP signaling is important for the development of the calyx of Held synapse *in-vivo* [8]. To obtain further evidence for a role of BMP signaling in calyx growth, we studied the effects of the BMP-receptor inhibitor LDN-193189 on the formation of large synapses. Organotypic slices from littermate *Math5*^*Cre*^ x *Brainbow* mice were cultured for 8 days either in the presence of LDN-193189 (5 μM) or in control conditions (DMSO alone), and then fixed and stained with anti-GFP and anti-Syt2 antibodies. We noted no obvious differences between the two conditions regarding the success rate of making slice cultures, nor in the survival and general quality of the cultured slices (Fig 5A and 5B). In the control organotypic cultures, we could once more identify large Math5—positive calyx-like synapses (Fig 5A''). However, in the presence of LDN-193189, Syt2- and Math5—positive nerve terminals in the MNTBs seemed strikingly smaller (Fig 5B", and inset).

We next quantified the nerve terminal size under the two conditions (Fig 5C). The distribution of nerve terminal sizes from the example organotypic slice shown in Fig 5A (control conditions) showed many nerve terminals with areas > 10 μm^2^, in agreement with the results of Fig 3 (Fig 5C; n = 21 / 68 nerve terminals were larger than 10 μm^2^). The organotypic slice cultured in parallel under LDN-193189 (Fig 5B) showed a conspicuous absence of large nerve terminals above 10 μm^2^ area (Fig 5C right; n = 38 nerve terminals). We next combined the distributions of nerve terminal size from n = 3 independent paired experiments (Fig 5D). This revealed a clear difference between LDN-193189 and control conditions, with a loss of nerve terminals in the size range of 5–50 μm^2^ in the presence of LDN-193189 (Fig 5D). This difference was also visible in cumulative histograms of the same data, which showed a leftward shift of nerve terminal size in the presence of LDN-193189 (Fig 5E). Correspondingly, the median nerve terminal areas were significantly smaller under LDN-193189 than in control conditions (Fig 5F; n = 3 cultures each; p = 0.0019). These effects were similar in type 1—and type 2 slice cultures (cross symbols and open circles in Fig 5F, respectively). When analyzed for all individual nerve terminals, the average nerve terminal area was 10.9 ± 0.63 μm^2^ in control slices (n = 559 nerve terminals from n = 3 slice cultures), and 4.7 ± 0.36 μm^2^ in the presence of LDN-193189 (n = 322 nerve terminals from n = 3 slice cultures; p < 0.001; Mann-Whitney test). This data thus shows that in the presence of the BMP—receptor inhibitor LDN-193189, nerve terminals developing *in-vitro* reach a significantly (~ 2-fold) smaller size than under control conditions.

## Discussion

We have established an organotypic slice culture model of growing calyx of Held nerve terminals which could facilitate future studies into the molecular mechanism of large nerve terminal growth. We used tissue from a *Math5*^*Cre*^ mouse line [22] to genetically label VCN bushy cells and their axons, a subset of which forms the large calyces of Held [21]. We found that an angle of ~15° during the preparation of coronal tissue slices significantly improved the preservation of the MNTB nuclei and of long GBC axons towards the midline. With these methods, we readily observed large, Math5—and Syt2- positive nerve terminals in the MNTB nucleus. These large nerve terminals must have formed *in-vitro*, because the cultures were prepared at P0, a time before the formation of the calyx of Held.

Previous work has shown that the superior olivary complex can be maintained in slice culture [26], but attempts to maintain the large calyx of Held nerve terminals in organotypic cultures have not been successful [17, 18]. Our study shows that three factors are critical for the success of organotypic cultures of the calyx of Held. First, we used tissue from newborn mice (P0), a time at which the large calyx of Held synapses are not yet present *in-vivo*. It is likely that plasticity of axonal reorganization present early after birth [37, 38] is beneficial for the growth of calyx—like nerve terminals in the cultured slices. Second, labelling bushy cells genetically by using the Math5 promoter [21, 22], has allowed us to reliably identify the VCN nucleus and bushy cells, and to visualize the length and position of bushy cell axons in organotypic culture *post-hoc*, and then to optimize the culturing conditions. Third, introducing a slice angle prevented axons from being cut too proximally.

We observed that Math5—positive bushy cells axons elaborated large calyx-like nerve terminals in the MNTB of organotypic cultures, whereas in the LSO, bushy cell axons exclusively had small nerve terminals (Fig 3). Saul et al. (2008) previously showed that Math5—positive axons innervate both the MNTB and the LSO (see also Fig 1). Math5—positive axons innervating the MNTB and the LSO most likely represent axons from GBCs and SBCs, respectively, in analogy to the GBC—SBC distinction found in cats [10, 11, 39]. Thus, our findings in organotypic culture suggest that the propensity of GBCs to elaborate large calyx of Held nerve terminals specifically onto MNTB neurons is preserved *in-vitro*.

A recent paper showed that calyx-like synapses can also form in dissociated cell cultures of auditory brainstem neurons of newborn mice [20]. This study used dissociated cell cultures of MNTB and VCN regions; VCN neurons were labelled by transfection with GFP. In co-cultures of the dissociated neurons, GFP—positive large calyx-like nerve terminals were observed onto GFP—negative neurons after 10 to 20 days *in-vitro*, when NT3, FGF2 and elevated K^+^ (25 mM) were added to the culturing medium [20]. In contrast, we did not add external growth factors, but similarly used high [K^+^] because this condition seems favorable for neuronal maintenance in cultures of auditory neurons [26]. The present (organotypic) and the previous dissociated culturing method each have advantages and limitations. The dissociated culture seems superior for imaging the presynaptic nerve terminal including time-lapse imaging [20]. On the other hand, the organotypic culture is better suited to preserve the tissue surrounding GBCs and their axons, including growth factors and cell-surface cues relevant for calyx of Held formation. Thus, the organotypic culture should be more amenable to loss-of-function approaches to identify molecular factors for calyx growth, as shown here by pharmacological inhibition of BMP signaling (Fig 5). On the other hand, the more reduced dissociated culture system will be better suited for gain-of-function approaches, exemplified by the necessity of adding NT3 and FGF2 to enable calyx growth *in-vitro* [20]. Both the present and the previous [20] culture study of the calyx of Held indicate that the intrinsic ability of GBCs to form large axo-somatic nerve terminals onto MNTB neurons is preserved *in-vitro*. This is consistent with the view that the formation of calyces is largely determined by intrinsic, genetically encoded factors, which likely include BMP- and NT3 signaling.

On the finer circuit- and connectivity level, the organotypic culture showed some important differences from the *in-vivo* situation. *In-vivo*, calyx of Held synapses form strictly on the contralateral MNTB [37, 40], whereas in the organotypic cultures large calyx-like synapses readily formed onto *ipsilateral* MNTB neurons. This was probably caused by the fact that in most cultures only a single VCN was preserved and therefore, the MNTB *ipsilateral* to the conserved VCN was denervated from its contralateral VCN input (see schemes in Fig 2). Interestingly, even when parts of the contralateral MNTB were preserved (type 2 organotypic cultures), we observed many large Math5—positive nerve terminals in the ipsilateral MNTB (Fig 4). These observations indicate that during the slicing procedure, many axons might be cut close to the *ipsilateral* MNTB. This, together with the degeneration of the axons from the contralateral side (see above), causes a situation which is similar to *in-vivo* lesion experiments of one VCN, in which calyx axons can re-organize and innervate the *ipsilateral* MNTB [37, 38].

Another difference to the *in-vivo* situation was that single Math5—positive axons were able to form several large nerve terminals onto several MNTB neurons (Fig 4). In contrast, *in-vivo*, 70% of the calyceal axons do not branch within the MNTB and elaborate only a single calyx [16]. The formation of multiple large synapses in the presence of few Math5—positive axons observed here *in-vitro* (Fig 4) indicates that *in-vivo*, competition between axons for a roughly equal number of target neurons helps to establish the 1: 1 connectivity in the calyx of Held pathway. Some axons nevertheless also branch *in-vivo* and make two calyces; this might be caused by a disparity in the number of GBCs as compared to MNTB neurons [16].

We demonstrated that the organotypic culture model of the calyx of Held is amenable to long-term pharmacological experiments, by showing that LDN-193189, an inhibitor of the phosphorylation of SMAD1, 5, 8 downstream of BMP-receptor activation [41], led to significantly smaller calyx—like synapses *in-vitro* (Fig 5). This adds pharmacological evidence for a role of BMP signaling in the development of the large calyx of Held synapse [8], and suggests that the classical BMP-signaling pathway (SMAD1, 5, 8 phosphorylation) [42] is involved in the growth program of calyces of Held. Genetic studies at the drosophila neuromuscular junction have indicated a role for BMP signaling in neuromuscular synapse growth, in a retrograde signaling direction of BMPs from the postsynaptic to the presynaptic compartment [43–45], but postsynaptic actions of BMPs were also shown [46]. Future work needs to establish whether the action of BMP at the calyx synapse is retrograde, or whether anterograde and/or autocrine signaling directions also contribute to the action of BMPs in calyx growth.

Investigating the physiological properties of synaptic transmission of large calyx—like synapses in organotypic culture has been beyond the scope of the present study. Note, however, that a previous study used organotypic cultures prepared from newborn mice, and obtained regular recordings of AMPA-receptor mediated miniature EPSC from putative MNTB neurons [19]. Thus, obtaining functional recordings in organotypic slice culture should be feasible. Nevertheless, we see the strength of the organotypic culture model not as a preparation for biophysical studies of presynaptic function, but rather, to enable studies into the molecular mechanisms of large nerve terminal growth, which can be observed by confocal microscopy *post-hoc* as demonstrated here.

### Conclusion

In summary, we describe an organotypic slice culture model in which calyx of Held synapses form *in-vitro*, and which allows to quantify large synapse size morphologically. The slice culture is amenable to long-term pharmacological manipulation. It should be similarly possible to apply methods of shRNA to further screen for molecular factors involved in calyx growth, and to use time-lapse imaging of XFP fluorescence in live preparations to observe axon re-organization and large nerve terminal growth more closely. Thus, the organotypic culture model of calyx of Held synapses could provide a useful new tool to study the molecular mechanisms specifying nerve terminal size in the mammalian brain.

## Supporting information

S1 Fig Initial attempts of organotypic slice cultures showed VCN and LSO, but could not preserve long Math5-positive axons or MNTB neurons. Example of a standard organotypic coronal slice culture obtained without an angle during slicing, using a *Math5*^*Cre*^ *x Brainbow* mouse. The slice was cultured at P0 and fixed after 9 DIV. ***A***, Confocal overview image of a slice stained with anti-GFP antibody (green channel), and with anti-VGluT2 antibody (red channel) as a marker for glutamatergic nerve terminals. Note the presence of Math5- (GFP) positive neurons in the VCN, and Math5- positive fibers in the LSO. ***B***, VCN at a higher magnification shows Math5-positive cells with typical bushy cell morphology. The dark spherical areas are occupied by GFP negative neurons. ***C***, Maximal intensity projection image (stack of n = 59 images taken at a z step of 0.5 μm) of the LSO area outlined in A. Note the presence of abundant Math5 (GFP)—positive axons. ***D***, Higher magnification image of the area outlined in C, showing VGluT2—positive small bouton-like nerve terminals, which often overlap with the Math5- positive axons. This indicates that bushy cell axons make small bouton-like synapses on the level of the LSO in organotypic cultures (see also Fig 3F).
(TIF)
