# Supplementary figures and images for: An organotypic slice culture to study the formation of calyx of Held synapses in-vitro
Source: PLoS One. 2017 Apr 18;12(4):e0175964. doi: 10.1371/journal.pone.0175964 (PMC5395213; doi:10.1371/journal.pone.0175964)

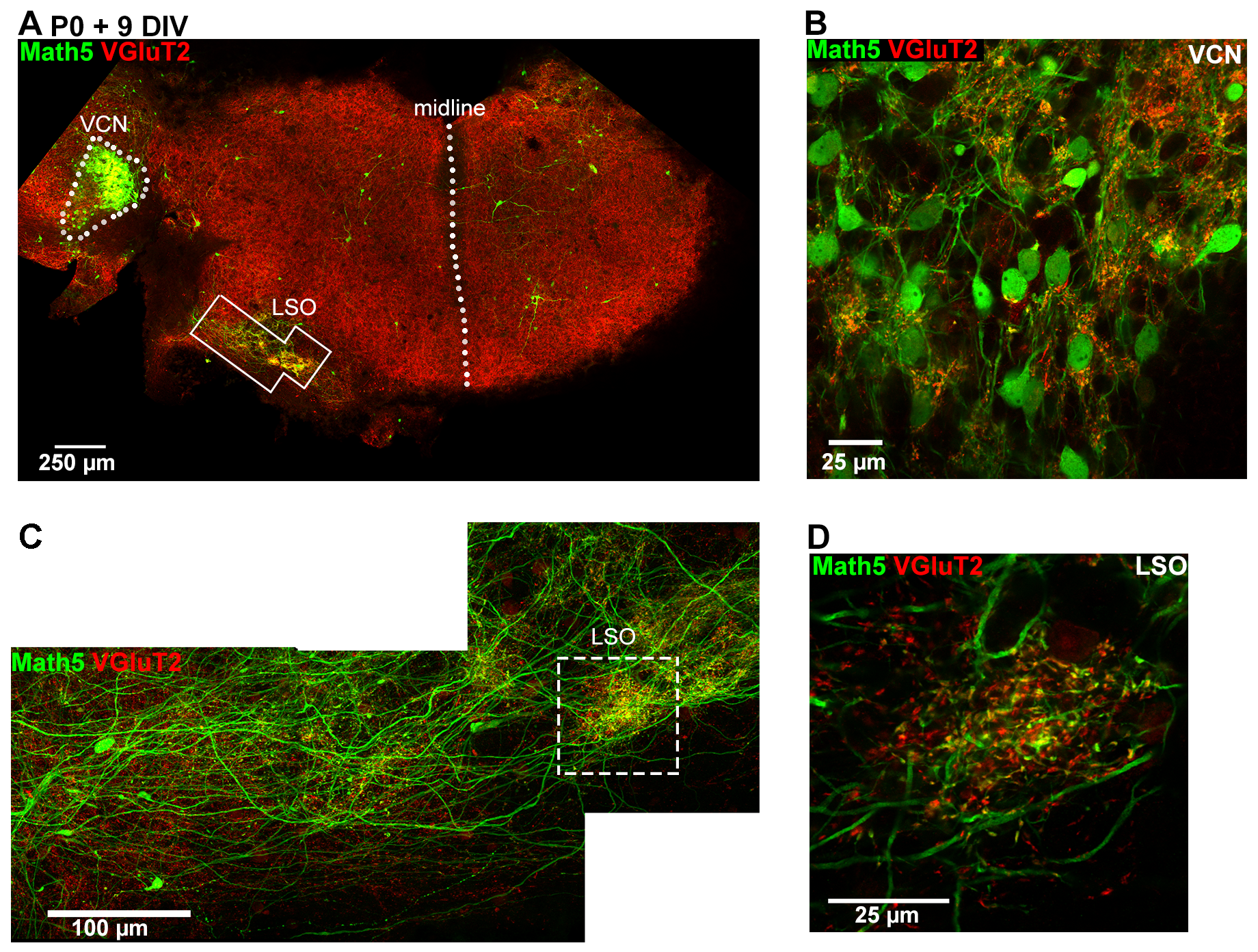

Supplement: S1 Fig — Example of a standard organotypic coronal slice culture obtained without an angle during slicing, using a Math5Cre x Brainbow mouse. The slice was cultured at P0 and fixed after 9 DIV. A, Confocal overview image of a slice stained with anti-GFP antibody (green channel), and with anti-VGluT2 antibody (red channel) as a marker for glutamatergic nerve terminals. Note the presence of Math5- (GFP) positive neurons in the VCN, and Math5- positive fibers in the LSO. B, VCN at a higher magnification shows Math5-positive cells with typical bushy cell morphology. The dark spherical areas are occupied by GFP negative neurons. C, Maximal intensity projection image (stack of n = 59 images taken at a z step of 0.5 μm) of the LSO area outlined in A. Note the presence of abundant Math5 (GFP)—positive axons. D, Higher magnification image of the area outlined in C, showing VGluT2—positive small bouton-like nerve terminals, which often overlap with the Math5- positive axons. This indicates that bushy cell axons make small bouton-like synapses on the level of the LSO in organotypic cultures (see also Fig 3F). (TIF) [file pone.0175964.s001.tif]
